# Rare Plasmid-Mediated AmpC Beta-Lactamase DHA-1 Located on Easy Mobilized IS26-Related Genetic Element Detected in *Escherichia coli* from Livestock and Food in Germany

**DOI:** 10.3390/microorganisms12030632

**Published:** 2024-03-21

**Authors:** Chiara Manfreda, Annemarie Kaesbohrer, Silvia Schmoger, Tanja Skladnikiewicz-Ziemer, Mirjam Grobbel, Alexandra Irrgang

**Affiliations:** 1 Department of Food and Drugs, University of Parma, 43126 Parma, Italy; chiara.manfreda@unipr.it; 2 Department Biological Safety, German Federal Institute for Risk Assessment, 10589 Berlin, Germanysilvia.schmoger@bfr.bund.de (S.S.);

**Keywords:** AmpC beta-lactamase, DHA-1, livestock, food, *E. coli*, Enterobacterales

## Abstract

AmpC beta-lactamases cause resistance to third-generation cephalosporins, including beta-lactamase inhibitors. In *Escherichia coli* from the German food production chain, the majority of AmpC beta-lactamase activity can be attributed to plasmid-mediated CMY-2 or overproduction of chromosomal AmpC beta-lactamase, but occasionally other enzymes like DHA-1 are involved. This study investigated the prevalence of the AmpC beta-lactamase DHA-1 in ESBL/AmpC-producing *E. coli* (*n* = 4706) collected between 2016 and 2021 as part of a German antimicrobial resistance monitoring program along the food chain. Eight isolates (prevalence < 0.2%) were detected and further characterized by PFGE, transformation and conjugation experiments as well as short-read and long-read sequencing. All eight strains harbored *bla*_DHA-1_ together with *qnrB4*, *sul1* and *mph(A)* resistance genes on an IS26 composite transposon on self-transferable IncFII or IncFIA/FIB/II plasmids. During laboratory experiments, activation of the translocatable unit of IS26-bound structures was observed. This was shown by the variability of plasmid sizes in original isolates, transconjugants or transferred plasmids, and correspondingly, duplications of resistance fragments were found in long-read sequencing. This activation could be artificial due to laboratory handling or naturally occurring. Nevertheless, DHA-1 is a rare AmpC beta-lactamase in livestock and food in Germany, and its dissemination will be monitored in the future.

## 1. Introduction

The increase in bacterial strains producing extended-spectrum beta-lactamases (ESBLs) and/or acquired AmpC beta-lactamases, which cause severe infections in humans, and their possible association with food or food-producing animals is of great concern to public health authorities [1,2].

Beta-lactamase production by Gram-negative bacteria, such as Enterobacterales, represents the main cause of beta-lactam resistance. Some of the beta-lactamases mediate resistance to third-generation cephalosporins, including oxyimino-cephalosporins (e.g., cefuroxime, cefotaxime, ceftriaxone, ceftazidime and cefepime), and are therefore referred to as ESBLs [3]. In contrast to AmpC beta-lactamases, ESBLs can often be inhibited by clavulanic acid or tazobactams.

Mobile elements, including conjugative plasmids, play a significant role in the transmission of ESBL enzymes in Enterobacterales, allowing rapid resistance dissemination. AmpC-mediated resistance in *Escherichia coli* can result either from constitutive overexpression of the chromosomal AmpC gene (due to mutations in the promoter/attenuator region) or from the acquisition of plasmid-mediated AmpC beta lactamases (pAmpC) [4].

There are different families of pAmpC: CMY-1 and -2, MIR, MOX, LAT, FOX, DHA, ACT, ACC and CFE [5]. All pAmpC enzymes belong to Ambler Class C, with CMY-2 being the most common in food-producing animals, particularly in broiler production [6]. Other pAmpCs are scarcely reported, one of which is DHA-1. This inducible AmpC beta-lactamase is closely related to the chromosomal AmpC gene of *Morganella morganii*, from which it is assumed to originate and successively migrate to plasmids [7,8,9,10]. It was first reported in *Salmonella enteritidis*, isolated from a clinical infection in Saudi Arabia in 1992 [11]. In principle, the Asian continent seems to have the highest prevalence of DHA-type enzymes in humans, associated mainly with *Klebsiella pneumoniae* [12,13]. Nevertheless, it has been globally detected in different Enterobacterales, such as *Klebsiella* spp., *Salmonella* spp. and *E. coli* [14,15,16,17,18,19,20,21,22].

In livestock animals, DHA-type enzymes were described in *Listeria monocytogenes* collected from cattle and their environment, including milking equipment at an Egyptian cattle farm in 2022, and in *S.* Heidelberg from cattle, pigs and turkey in the USA in 2009 [23,24]. Recent publications have also described the presence of the *bla*_DHA-1_ gene in *E. coli* and *Citrobacter freundii* in frozen chicken carcasses from Mozambique, Brazil and South Africa and several *K. pneumoniae* and one *E. coli* in chicken fecal samples from Malaysia [25,26]. Further, *bla*_DHA-1_ was detected in Enterobacterales, such as *Proteus mirabilis*, *E. coli* and *K. pneuomoniae*, collected from pig production in India and Korea [12,27]. Food of animal origin can also be considered a source of *bla*_DHA-1_ as it has been described in a number of publications in recent years, especially in *Pseudomonas aeruginosa* collected from bovine and ovine meat, raw milk and fishery products from Iran [28,29,30,31] but also in *K. pneumoniae* and *E. coli* strains isolated from shrimp in Germany, in *E. coli* isolated from beef products in South Africa and in *K. pneumoniae* isolated from ready-to-eat sandwiches in Algeria [32,33,34].

Although DHA-1-producing bacteria have been found in the food production chain, data on their prevalence in livestock and food is missing. The aim of this study was to assess the occurrence of *bla*_DHA-1_ in *E. coli* isolates collected as part of antimicrobial resistance (AMR) monitoring of the German food production chain and to characterize them genetically.

## 2. Materials and Methods

### 2.1. Sampling and Isolation of E. coli

For this study, ESBL/AmpC-producing *E. coli* isolates from the German antimicrobial resistance monitoring program for the years 2016–2021 were investigated. The AMR monitoring was conducted following technical specifications given by Commission Implementing Decision (CID) 2013/652/EU and CID 2020/1729/EU and complemented by additional national programs using the same methods [35,36]. The samples were taken from feces at primary production, caecum content at slaughter or food at retail by the federal states and investigated at the regional laboratories. Indicator *E. coli* samples were sent to the national reference laboratory for antimicrobial resistance (NRL-AR) at the German Federal Institute for Risk Assessment (BfR) and subjected to antimicrobial susceptibility testing. From the same samples, selective isolation of ESBL/AmpC-producing *E. coli* was conducted according to the protocol provided by the European Reference Laboratory for Antimicrobial Resistance (EURL-AR; https://www.eurl-ar.eu/protocols.aspx (accessed on 19 December 2023)). In brief, each sample was diluted 1:10 with buffered peptone water, homogenized and incubated overnight for 18–24 h at 37 °C. A 10 µL loop was streaked out on MacConkey agar supplemented with 1 μg/mL cefotaxime for 18–24 h at 37 °C. *E. coli* isolates were confirmed by MALDI-TOF (Bruker Daltonik GmbH, Leipzig, Germany).

### 2.2. Antimicrobial Susceptibility Testing

In the years 2014–2020, all *E. coli* isolates were tested for their susceptibility to 14 different antimicrobials by determination of the minimal inhibitory concentration (MIC) using broth microdilution with commercial plates (Thermo Fisher Scientific, Schwerte, Germany) following CID 2013/652/EU [35]. All isolates with resistance to cefotaxime and/or ceftazidime were tested on a second MIC panel for phenotypic characterization of the beta-lactamase (Thermo Fisher Scientific, Schwerte, Germany). Since 2021, commensal *E. coli* samples have been phenotypically characterized by MIC according to CID 2020/1729/EU with the antimicrobial panel EUVSEC3 (Thermo Fisher Scientific, Schwerte, Germany) [36]. Following this CID indicator, *E. coli* resistant to cefotaxime and/or ceftazidime, as well as selectively resistant to cefotaxime, were isolated and subjected to whole-genome sequencing (WGS). MIC values were interpreted using ECOFF values given in the CID or, when missing in the CID, defined by the European Food Safety Authority (EFSA). In this study, non-wild-type isolates with an MIC value above ECOFF are referred to as “resistant”. As antimicrobial panel and ECOFF values differ between CIDs, all *bla*_DHA-1_-positive isolates were tested with the new panel (EUVSEC3) and interpreted with the actual ECOFF values.

### 2.3. Molecular Analysis

All phenotypically ESBL/AmpC-suspicious isolates (*n* = 4706) between 2016 and 2020 were investigated by real-time PCR for the presence of *bla*_CTX_, *bla*_TEM_, *bla*_SHV_ and *bla*_CMY_ genes according to Roschanski et al. (2014) prior to this study [37]. Isolates with a MIC profile suspicious for AmpC production and *bla*_CMY_-negative by real-time PCR were investigated by PCR for the presence of DHA family-specific plasmid-mediated AmpC genes (*bla*_DHA-1_, *bla*_DHA-2_). PCR was also performed for ESBL-suspicious isolates for which no other ESBL genes were detected. In sum, 410 *E. coli* isolates were screened. The primers used for the amplification were DHA-F (5′-AACTTTCACAGGTGTGCTGGGT-3′) and DHA-R (5′- CCGTACGCATACTGGCTTTGC-3′), with an annealing temperature of 60 °C and an expected amplicon size of 405 bp [38]. Since 2021, all cefotaxime-resistant isolates have been investigated by WGS, including AMR gene detection with AMRFinder (please see below).

All *bla*_DHA-1_-positive isolates were characterized phylogenetically by XbaI pulsed-field gel electrophoresis (PFGE) using the PulseNet PFGE protocol (https://www.cdc.gov/pulsenet/pdf/ecoli-shigella-salmonella-pfge-protocol-508c.pdf; accessed on 19 December 2023). The localization of the *bla*_DHA-1_ gene in plasmids was determined by digestion with S1 nuclease with subsequent Southern blot hybridization using a *bla*_DHA-1_ probe [39].

From each isolate, plasmids were extracted using the Agencourt CosMCPrep Kit (Beckman Coulter, Krefeld, Germany) according to the manufacturer’s instructions. The transformation process was carried out using GeneHogs^®^ *E. coli* strain as a competent cell and 0.1 cm disposable cuvettes in a MicroPulser electroporator (both from Bio-Rad, Feldkirchen, Germany) according to the manufacturer’s instructions. Cells were recovered in SOC medium for 1 h at 37 °C, and 100 µL was plated on LBA supplemented with 1 mg/L cefotaxime for 48 h at 37 °C. Up to 4 colonies were picked, and the transformation of DHA-1-harboring plasmid was confirmed by PCR as described above. The size of the transferred plasmid was determined by S1-PFGE. Further, plasmid replicon type was determined by PCR replicon typing using PBRT 2.0 (Diatheva, Fano, Italy). Transferability of the *bla*_DHA-1_-harboring plasmids was investigated by filter mating experiments at 37 °C with the original strains as well as the transformed cells as donors and the *E. coli* J53 strain as recipients [40].

### 2.4. Sequencing Analysis

WGS of the *bla*_DHA-1_-positive isolates was performed. Genomic DNA was isolated from overnight cultures using the PureLink**^®^** Genomic DNA Mini Kit (Thermo Fisher Scientific, Schwerte, Germany). For Illumina NextSeq short-read sequencing, libraries were prepared with the Nextera™XT DNA Library Preparation Kit or the Nextera™DNA Flex Library Preparation Kit (Illumina, San Diego, CA, USA) according to the manufacturer’s protocol. Raw reads were quality checked and processed using the AQUAMIS pipeline (https://gitlab.com/bfr_bioinformatics/AQUAMIS (accessed on 19 December 2023)) [41].

Long-read sequencing was performed using MinIon Mk1C with Rapid Barcoding Kit 96 (SQK-RBK110.96) and an R9.4.1 flow cell (ONT—Oxford Nanopore Technologies, Oxford, UK). Long-read data were processed by the MiLongA pipeline (https://gitlab.com/bfr_bioinformatics/milonga (accessed on 19 December 2023)), which included the hybrid assemblers Flye (2.9-b1768) and Unicycler (v0.4.8). For further analysis, Unicycler hybrid assemblies were mainly used. Assemblies were analyzed by our in-house pipeline Bakcharak (https://gitlab.com/bfr_bioinformatics/bakcharak (accessed on 19 December 2023)) with regard to multi-locus sequence typing (MLST), Clermont typing, AMR genes, plasmid typing or serotyping. Additionally, web-based tools of the Center for Genomic Epidemiology (http://www.genomicepidemiology.org (accessed on 18 December 2023)) were used for comparative analyses: MINTyper v.1.0 [42] with default settings for SNP analysis and cgMLSTFinder 1.2 version 1.0.1 [43,44]. Additionally, the chewieSnake pipeline (https://gitlab.com/bfr_bioinformatics/chewieSnake (accessed on 19 December 2023)) was used for core genome MLST (cgMLST) [45]. Annotation was carried out using RASTk on PATRIC (www.bv-brc.org (accessed on 19 December 2023)), and ISfinder was used for identification of mobile genetic elements (https://www-is.biotoul.fr (accessed on 19 December 2023)). BRIG (v.0.95) was used to display the mapping of reads against reference plasmids.

Raw short-read sequences were deposited at NCBI under BioProject accession number PRJNA995946 with SRA accession number SRR25318555-62, as were the complete *bla*_DHA-1_ plasmid sequences of 19-AB01500 (pEC19-AB01500 Genbank accession no. OR347004), 19-AB02469 (pEC19-AB02469 Genbank accession no. OR351271) and 21-AB01800 (pEC21-AB01800 Genbank acc. no. PP129611) as prototypes for the detected plasmids.

## 3. Results and Discussion

Between 2016 and 2021, a total of 4706 ESBL/AmpC-producing *E. coli* were detected within the German AMR monitoring program. According to the study criteria, 410 isolates from the years 2016–2020 were screened by PCR for the presence of *bla*_DHA_, whereas in 2021, ESBL/AmpC determination was conducted by WGS. In total, eight *bla*_DHA-1_-producing *E. coli* were detected in livestock and food in Germany. In particular, four were obtained from pig production (primary production or at slaughter), two were obtained from freshwater fish (*Pangasianodon* sp.; presumably both imported), one was collected from a calf at slaughter and one was recovered from wildlife (wild goose feces).

According to the high number of investigated ESBL/AmpC-producing isolates in total within the AMR monitoring program (*n* = 4706), *bla*_DHA-1_ can be considered a rare AmpC genotype in the German food production chain with a prevalence below 0.2%. This is comparable to other European countries, where the prevalence of *bla*_DHA-1_ in food-producing animals and meat was between 0% and 1% in 2021 in the countries reporting WGS data [46]. Although DHA-1-producing bacteria can be found in livestock and food, the food chain does not seem to be the main reservoir. Most reports can be found from human infections, with an emphasis on *Klebsiella* spp. in Asian countries [12,13].

Among the eight investigated isolates in this study, five different MIC profiles were found. In general, the isolates were resistant to six to seven different antimicrobial classes. Thus, all were multidrug resistant, exhibiting resistance to ampicillin, azithromycin, ciprofloxacin, cefotaxime and ceftazidime. Moreover, the majority of isolates (6/8) showed potential pAmpC presence, while the other isolates (2/8) were classified as a combination suspicious for ESBL/pAmpC (Table 1). This was consistent with the genotypes, as these two isolates harbored additional *bla*_CTX-M_ genes.

**Figure 1:**
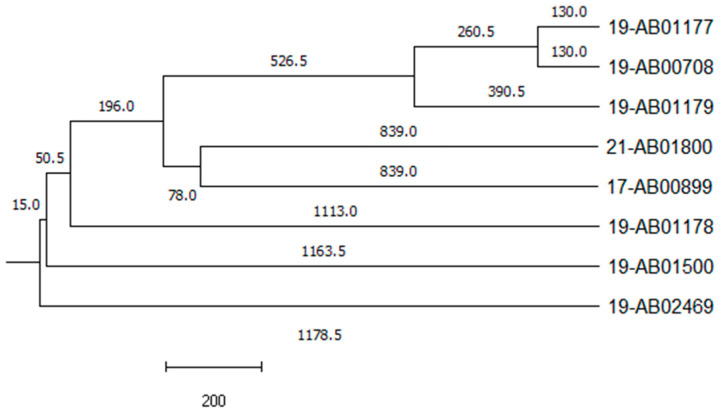
Phylogenetic relationship of DHA-1-producing *E. coli*. Single linkage tree based on cgMLST analysis. The numbers on the branches indicate the number of allele differences.

The phylogenetic relationship of the isolates was investigated by PFGE and SNP analysis (Supplementary Table S1) using Illumina raw reads and cgMLST using assembled genomes. All three methods revealed a non-clonal phylogenetic relationship. The single linkage trees of SNP analysis and cgMLST (Figure 1) were highly similar. This is in concordance with the high diversity of the five different phylogenetic groups of the isolates, the different matrices from which the isolates originate and the long period of 5 years in which these eight positive samples were taken. An epidemiological link could have been possible for isolates 19-AB01177, 19-AB01178 and 19-AB01179 as the samples were taken at the same time from the same region, but it was not observed during the analysis.

Because no clonal relationship was detected, a spread of *bla*_DHA-1_ via plasmids was hypothesized. The *bla*_DHA-1_-harboring plasmid was self-transferable by conjugation, and the gene was transferable by transformation. Based on PBRT replicon typing, the gene was located on IncFII or IncFIA/FIB/FII plasmids (Table 2). Determination of the plasmid size and its characteristics proved to be difficult by molecular methods. S1-PFGE as well as subsequent Southern blot hybridization revealed no clear plasmid determination for three of the eight isolates. Transformation of the *bla*_DHA-1_-harboring plasmids also revealed inconsistent results for four isolates (Table 2). Different transferred plasmids from the same original isolates showed different sizes in S1-PFGE, which also did not correspond to the sizes of plasmids detected in the original isolates. Similar problems were found with transconjugation.

There are different possible explanations for the inconclusive results. The first option is that there was more than one copy of the *bla*_DHA-1_ gene present on different plasmids. The second option is that the *bla*_DHA-1_ gene is part of a literally mobile genetic element that was mobilized during laboratory handling, leading to artificially altered results. Comprehensive genomic investigations with short- and long-read sequencing approaches were conducted to obtain a closer insight into the genetic structure of *bla*_DHA-1_ plasmids.

**Figure 2:**
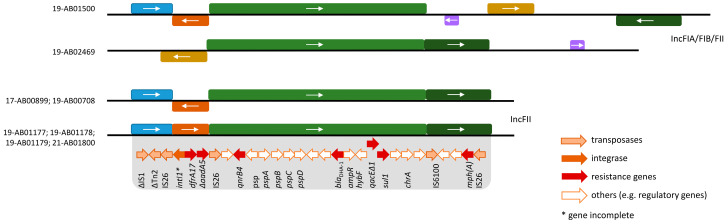
Genetic organization of the *bla*_DHA-1_ region. The *bla*_DHA-1_ gene is located on an IS26 composite transposon with the same structure in all isolates, except for 19-AB01500, in which additional resistances were incorporated. Flanking sequences of the IS26 composite transposon showed variabilities. The same colors in the boxes indicate the same genetic structures. Orientation of the gene fragments according to the position under (−) or above (+) the line is indicated by an arrow. The illustration is schematic and not to scale.

**Figure 3:**
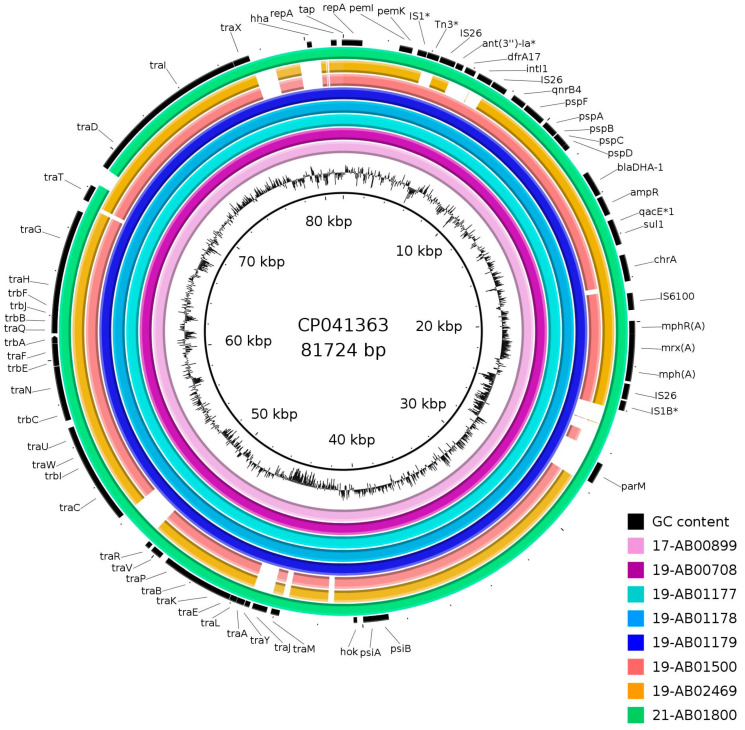
Mapping of *bla*_DHA-1_-harboring plasmids in livestock and food in Germany against IncFII plasmid p133355_SW_C4_Cam-1 in *Citrobacter amalonaticus* (NZ_CP041363); genes with an asterisk are truncated or incomplete in the reference.

Assemblies of Illumina short-read sequences revealed a 15,141–15,143 bp long circular fragment, which was flanked by 91 bp inverted repeats and harbored resistance genes against cephalosporins (*bla*_DHA-1_), fluoroquinolones (*qnrB4*), sulfonamides (*sul1*) and macrolides (*mph(A)*). Hybrid assemblies of long- and short-read sequences could extend this to 18,322–19,970 bp long fragments. There, the fragment found in short-read sequences was flanked by two IS26 element transposases, forming a composite transposon (Figure 2). This was associated with an additional incomplete class I integrase, the trimethoprim resistance gene *dfrA17*, the truncated aminoglycoside resistance gene *aadA5* and another IS26 transposase, except for isolate 19-AB02469 (Figure 2). This structure was found to have very high similarities to the IncFII plasmid p133355_SW_C4_Cam-1 in *Citrobacter amalonaticus* (NZ_CP041363) from humans and pIV_IncFII_DHA in *E. coli* (MN537908) from an infected dog [47,48]. Mapping of the Illumina raw reads against NZ_CP041363 (Figure 3) and MN537908 also revealed high similarities to the plasmid backbone. Inversion of the *int1-dfrA17-aadA5* fragment was found in three of the isolates confirmed by PCR. Closed plasmids were obtained by long-read sequencing for only three isolates. Two of these plasmids showed a divergent flanking structure of the *bla*_DHA-1_-harboring fragment (Figure 2). The high density of mobile genetic elements, especially repeated IS26 transposases in the region, hampered complete plasmid assembly for the remaining isolates.

Further, the mappings showed that the *bla*_DHA-1_-harboring fragment had a higher coverage by the reads than the plasmid average, suggesting that more than one copy might be possible. Additionally, inverted repeats flanking IS26 transposase were at least found in three further positions on the plasmid of 19-AB02469, where long-read sequencing revealed a complete 160 kb long plasmid sequence. This suggested that insertion and excision events of IS26 elements had taken place in the past and that mobilization during laboratory work was likely. For confirmation of this hypothesis, different transformed cells in the samples were investigated by long-read sequencing. With this, we could resolve some of the issues by taking into account both assembly strategies (Flye and Unicycler). Although Flye tends to duplicate small plasmids, it mostly showed duplications of resistance genes when the transferred plasmid also showed a bigger size in S1-PFGE compared to other transferred plasmids of the same transformation experiments and the same original strain [49]. The copied resistance fragment is supported by much higher coverage values compared to the rest of the plasmid. Additionally, for 19-AB00708 and 19-AB01179, some *bla*_DHA-1_-transformed plasmids showed a two- or three-times larger size than that estimated by the sequencing data. Therefore, in addition to *bla*_DHA-1_ fragment duplications via lab handling, complete plasmid copying also took place.

As it is not possible to determine the physiological sizes and structure of the *bla*_DHA-1_ plasmids, except for 19-AB01500 and 19-AB02469, the plasmid sizes given in Table 2 should be understood as the minimum plasmid sizes. These result from the sum of the *bla*_DHA-1_ fragment and the size of the plasmid backbone.

We hypothesize that plasmid size variabilities were artificial and due to lab handling. Although it could theoretically also occur in bacterial cells in the natural habitat, the low prevalence of ESBL/AmpC-producing *E. coli* in the German food chain, as well as the reported highly similar plasmids in Enterobacterales from humans and animals from different countries and different years, does not support a naturally highly active mobile genetic element [47,48]. The structure found in animals with resistance islands harboring *∆aadA5*, *dfrA17*, *qnrB4*, *bla*_DHA-1_ and *mph(A)* seems to be stable but is not frequently reported in humans. Further, most of the reported *bla*_DHA-1_-producing bacteria were obtained from human infections associated with *Klebsiella* sp. [50]. However, as the ESBL/AmpC monitoring in livestock and food is limited to *E. coli*, we cannot estimate the spread of DHA-1 in other Enterobacterales and the associated genetic structure. However, most *bla*_DHA-1_ plasmids are self-transmissible, and if there was a spread of DHA-1-producing Enterobacterales, a higher prevalence in *E. coli* would also be expected as long as the related plasmids are able to replicate in *E. coli*. In the reported Enterobacterales from humans, a variety of plasmids (IncF, IncHI2, IncR, IncL/M and IncA/C_2_) of different sizes and co-resistance genes have been reported [14,18,48,51]. In all species and matrices, *bla*_DHA-1_ is associated with *qnrB4*. Typically, further ESBL genes are detected in human samples, whereas only two of the isolates in this study harbored additional *bla*_CTX-M_ genes [9,15,16,18,50].

A big driver for resistance spread is IS26 [52]. IS26 transposons can be complex, and several transposons can overlap. Transition of IS26 with incorporated (resistance) genes in conservative mode includes the formation of translocatable units (TU), and it is more efficient when another IS26 is recognized and insertion takes place next to it [53]. The formation of such cointegrates was observed in this study. The excision of TUs leaves an IS26 behind. This explains the high density of IS26 all over the *bla*_DHA-1_ plasmids [54]. As IS26 is located in different orientations, there might be an overlap of IS26-bound structures, as shown for Tn1696-related transposons in IncHI2 plasmids [55].

## 4. Conclusions

*Bla*_DHA-1_ is prevalent but rare in livestock and food in Germany. Although the gene was located in self-transferable plasmids and in active IS26-bounded structures, the spread of the gene seems to be limited. Ecological niches for related plasmids might be covered by other ESBL/pAmpC-gene-harboring plasmids of *E. coli*, or the spread of *bla*_DHA-1_ takes place in other Enterobacterales than those that were monitored. As dissemination can change over time due to varying environmental conditions, trade networks and food production systems, ongoing monitoring of ESBL/pAmpC-producing Enterobacterales is needed to assess the potential of consumers being exposed to multidrug-resistant bacteria. Situations where such resistance genes find their niche and spread within a production chain need to be detected early. Although resistance genes themselves are not pathogenic, their spread to potentially pathogenic species can limit the therapeutic options in cases of infection.

This study shows that molecular methods or sequencing analyses alone are not enough to reveal the genetic organization of some bacterial strains and their resistance determinants, and a combination of different approaches is required to evaluate the potential for increasing or emerging public health risks.

## Figures and Tables

**Table 1:** Isolate characteristics of DHA-1-producing *E. coli* from the food production chain in Germany 2016–2021.

Isolate ID	Matrix	Resistance Profile ^1^ [Beta-Lactam Phenotype ^2^]	Phylogenetic Group	MLST	Resistance Genes ^3^
17-AB00899	Calves, feces	AMP, AZI, CIP, FOT, NAL, TAZ, TET, TMP [AmpC]	B1	446	*bla*_DHA-1_; *dfrA17*; *mph(A)*; *qnrB4*; *su*l*1*; *tet(A)*; *tet(B)*
19-AB00708	Fattening pig, feces	AMP, AZI, CIP, FOT, NAL, TAZ, TET, TMP [AmpC]	C	23	*bla*_DHA-1_; *dfr*A17; *mph*(A); *qnr*B4; *sul*1; *tet(A)*
19-AB01177	Fattening pig, feces	AMP, AZI, CIP, FOT, NAL, SMX, TAZ, TET, TMP [AmpC]	C	369	*bla*_DHA-1_; *bla*_TEM-1_; *dfrA1*; *dfrA17; mph(A); qnrB4; sul1; sul2; tet(A)*
19-AB01178	Fattening pig, feces	AMP, AZI, CIP, FOT, NAL, SMX, TAZ, TET, TMP [AmpC]	E	118	*aadA1; aph(3’)-Ia; bla*_DHA-1_; *bla*_TEM-34_; *dfrA1; dfrA17; emrD; mph(A); qnrB4; sat2; sul1; sul2; tet(B)*
19-AB01179	Fattening pig, feces	AMP, AZI, CIP, FOT, NAL, SMX, TAZ, TET, TMP [AmpC]	C	5271	*aadA1; aph(3’’)-Ib; aph(6)-Id; bla*_DHA-1_, *bla*_TEM-1_; *dfrA1; dfrA17; mph(A); qnrB4; sul1; sul2; tet(A)*
19-AB01500	Freshwater fish	AMP, AZI, CIP, FOT, NAL, SMX, TAZ, TET, TMP [ESBL/AmpC]	D	69	*aph(3’’)-Ib; aph(6)-Id; bla*_CTX-M-27_; *bla*_DHA-1_; *bla*_TEM-1_; *cyaA*_S352T; *dfrA14; dfrA17*; *erm*(B) *; *gyrA*_S83L; *mdtM*; *mph*(A); *parE*_D476N; qnrB4; sul1; sul2; tet(B) *
19-AB02469	Wild goose, feces	AMP, AZI, CIP, FOT, NAL, SMX, TAZ, TET [ESBL/AmpC]	B2	131	*aph(3’’)-Ib; aph(6)-Id; bla*_CTX-M-15_; *bla*_DHA-1_; *gyr*A_D87N; *gyrA*_S83L; *mph(A)*; *parC*_E84V; *parC*_S80I; *parE*_I529L; *ptsI*_V25I; *qnrB4; sul1; sul2, tet(A)*; *uhpT*_E350Q
21-AB01800	Freshwater fish	AMP, AZI, CIP, FOT, SMX, TAZ, TMP [AmpC]	B1	3570	*bla*_DHA-1_; *bla*_TEM-1_; *dfr*A17; *mph*(A); *qnrB4*; *sul1*

^1^ Resistance profile is based on MIC data from the first panel. ^2^ Beta-lactam phenotype was determined by the MIC data of the second panel. ^3^ AMR genes obtained by WGS; genes with less than 100% identity to reference (*) were only listed when there was a corresponding phenotype, except for multidrug-resistant transporters (<100% identity not included). Abbreviations: AMP: ampicillin; AZI: azithromycin; CIP: ciprofloxacin; FOT: cefotaxime; NAL: nalidixic acid; TAZ: ceftazidime; SMX: sulfamethoxazole; TET: tetracycline; TMP: trimethoprim.

**Table 2:** Characterization of *bla*_DHA-1_-harboring plasmids.

Isolate	Plasmid Size (in kb)		Incompatibility Group	Conjugative
	PFGE, Transformed Plasmid	WGS ^1^		
17-AB00899	92/74	83.5	FII	yes
19-AB00708	210/193	82	FII	yes
19-AB01177	87	77	FII	yes
19-AB01178	75	82	FII	yes
19-AB01179	141/178/88/148	78.5	FII	yes
19-AB01500	141	149	FII, FIA, FIB	yes
19-AB02469	152	160	FII, FIA, FIB	yes
21-AB01800	58/110	63.5	FII	yes

^1^ Minimum plasmid size, obtained from Unicycler assemblies.

## Data Availability

Raw sequencing data and complete reference plasmids are deposited at NCBI (see Section 2.3).

## Supplementary Materials

The following supporting information can be downloaded at: https://www.mdpi.com/article/10.3390/microorganisms12030632/s1, Table S1: SNP analysis of DHA-1-producing *E. coli* from the food chain in Germany.

## References

[1] European Food Safety Authority (2011). Scientific Opinion on the public health risks of bacterial strains producing extended-spectrum β-lactamases and/or AmpC β-lactamases in food and food-producing animals. EFSA J..

[2] European Centre for Disease Prevention and Control (2014). Systematic Review of the Effectiveness of Infection Control Measures to Prevent the Transmission of Carbapenemase-Producing Enterobacteriaceae through Cross-Border Transfer of Patients.

[3] Livermore D. (2009). β -Lactamases- the threat renews. Curr. Protein Pept. Sci..

[4] Peter-Getzlaff S., Polsfuss S., Poledica M., Hombach M., Giger J., Böttger E.C., Zbinden R., Bloemberg G.V. (2011). Detection of AmpC beta-lactamase in *Escherichia coli*: Comparison of three phenotypic confirmation assays and genetic analysis. J. Clin. Microbiol..

[5] Jacoby G.A. (2009). AmpC beta-lactamases. Clin. Microbiol. Rev..

[6] Bush K., Jacoby G.A. (2010). Updated Functional Classification of β-Lactamases. Antimicrob. Agents Chemother..

[7] Reisbig M.D., Hanson N.D. (2002). The ACT-1 plasmid-encoded AmpC beta-lactamase is inducible: Detection in a complex beta-lactamase background. J. Antimicrob. Chemother..

[8] Rayamajhi N., Jung B.Y., Cha S.B., Shin M.K., Kim A., Kang M.S., Lee K.M., Yoo H.S. (2010). Antibiotic resistance patterns and detection of *bla*_DHA-1_ in *Salmonella* species isolates from chicken farms in South Korea. Appl. Environ. Microbiol..

[9] Pai H., Kang C.-I., Byeon J.-H., Lee K.-D., Park W.B., Kim H.-B., Kim E.-C., Oh M., Choe K.-W. (2004). Epidemiology and clinical features of bloodstream infections caused by AmpC-type-β-lactamase-producing *Klebsiella pneumoniae*. Antimicrob. Agents Chemother..

[10] Barnaud G., Arlet G., Verdet C., Gaillot O., Lagrange P.H., Philippon A. (1998). *Salmonella enteritidis*: AmpC plasmid-mediated inducible β-Lactamase (DHA-1) with an ampR gene from *Morganella morganii*. Antimicrob. Agents Chemother..

[11] Gaillot O., Clément C., Simonet M., Philippon A. (1997). Novel transferable beta-lactam resistance with cephalosporinase characteristics in *Salmonella enteritidis*. J. Antimicrob. Chemother..

[12] Rayamajhi N., Kang S., Lee D., Kang M., Lee S., Park K., Lee H., Yoo H. (2008). Characterization of TEM-, SHV- and AmpC-type β-lactamases from cephalosporin-resistant Enterobacteriaceae isolated from swine. Int. J. Food Microbiol..

[13] Lee K., Lee M., Shin J.H., Lee M.H., Kang S.H., Park A.J., Yong D., Chong Y. (2006). Prevalence of plasmid-mediated AmpC β -lactamases in *Escherichia coli* and *Klebsiella pneumoniae* in Korea. Microb. Drug Resist..

[14] Ribeiro T.G., Novais Â., Rodrigues C., Nascimento R., Freitas F., Machado E., Peixe L. (2019). Dynamics of clonal and plasmid backgrounds of Enterobacteriaceae producing acquired AmpC in Portuguese clinical settings over time. Int. J. Antimicrob. Agents.

[15] Kis Z., Tóth Á., Jánvári L., Damjanova I. (2016). Countrywide dissemination of a DHA-1-type plasmid-mediated AmpC β-lactamase-producing *Klebsiella pneumoniae* ST11 international high-risk clone in Hungary, 2009–2013. J. Med. Microbiol..

[16] Freitas F., Machado E., Ribeiro T.G., Novais Â., Peixe L. (2014). Long-term dissemination of acquired AmpC β-lactamases among *Klebsiella* spp. and *Escherichia coli* in Portuguese clinical settings. Eur. J. Clin. Microbiol. Infect. Dis..

[17] Empel J., Hrabák J., Kozińska A., Bergerová T., Urbášková P., Kern-Zdanowicz I., Gniadkowski M. (2010). DHA-1-producing *Klebsiella pneumoniae* in a teaching hospital in the Czech Republic. Microb. Drug Resist..

[18] Diestra K., Miró E., Martí C., Navarro D., Cuquet J., Coll P., Navarro F. (2011). Multiclonal epidemic of *Klebsiella pneumoniae* isolates producing DHA-1 in a Spanish hospital. Clin. Microbiol. Infect..

[19] Estaleva C.E.L., Zimba T.F., Sekyere J.O., Govinden U., Chenia H.Y., Simonsen G.S., Haldorsen B., Essack S.Y., Sundsfjord A. (2021). High prevalence of multidrug resistant ESBL- and plasmid mediated AmpC-producing clinical isolates of *Escherichia coli* at Maputo Central Hospital, Mozambique. BMC Infect. Dis..

[20] Liebana E., Batchelor M., Clifton-Hadley F.A., Davies R.H., Hopkins K.L., Threlfall E.J. (2004). First report of *Salmonella* isolates with the DHA-1 AmpC beta-lactamase in the United Kingdom. Antimicrob. Agents Chemother..

[21] Moland E.S., Black J.A., Ourada J., Reisbig M.D., Hanson N.D., Thomson K.S. (2002). Occurrence of newer β-Lactamases in *Klebsiella pneumoniae* isolates from 24 U.S. hospitals. Antimicrob. Agents Chemother..

[22] Alvarez M., Tran J.H., Chow N., Jacoby G.A. (2004). Epidemiology of conjugative plasmid-mediated AmpC β-Lactamases in the United States. Antimicrob. Agents Chemother..

[23] Lynne A.M., Kaldhone P., David D., White D.G., Foley S.L. (2009). Characterization of antimicrobial resistance in *Salmonella enterica* serotype Heidelberg isolated from food animals. Foodborne Pathog. Dis..

[24] Elsayed M.M., Elkenany R.M., Zakaria A.I., Badawy B.M. (2022). Epidemiological study on *Listeria monocytogenes* in Egyptian dairy cattle farms’ insights into genetic diversity of multi-antibiotic-resistant strains by ERIC-PCR. Environ. Sci. Pollut. Res..

[25] Tan H.-S., Pan Y., Agustie H., Loh H.-S., Rayamajhi N., Fang C.-M. (2023). Characterisation of ESBL/AmpC-Producing Enterobacteriaceae isolated from poultry farms in Peninsular Malaysia. Lett. Appl. Microbiol..

[26] Faife S.L., Zimba T., Sekyere J.O., Govinden U., Chenia H.Y., Simonsen G.S., Sundsfjord A., Essack S.Y. (2020). β-lactam and fluoroquinolone resistance in Enterobacteriaceae from imported and locally-produced chicken in Mozambique. J. Infect. Dev. Ctries..

[27] Chinnam B.K., Nelapati S., Tumati S.R., Bobbadi S., Chaitanya Peddada V., Bodempudi B. (2021). Detection of β-lactamase–producing *Proteus mirabilis* strains of animal origin in Andhra Pradesh, India and their genetic diversity. J. Food Prot..

[28] Rezaloo M., Motalebi A., Mashak Z., Anvar A. (2022). Prevalence, antimicrobial resistance, and molecular description of *Pseudomonas aeruginosa* isolated from meat and meat products. J. Food Qual..

[29] Poursina S., Ahmadi M., Fazeli F., Ariaii P. (2023). Assessment of virulence factors and antimicrobial resistance among the *Pseudomonas aeruginosa* strains isolated from animal meat and carcass samples. Vet. Med. Sci..

[30] Dehkordi S.M.H., Anvar S.A., Rahimi E., Ahari H., Ataee M. (2022). Prevalence, phenotypic and genotypic diversity, antibiotic resistance, and frequency of virulence genes in *Pseudomonas aeruginosa* isolated from shrimps. Aquacult Int..

[31] Dehkordi S.M.H., Anvar S.A., Rahimi E., Ahari H., Ataee M. (2022). Molecular investigation of prevalence, phenotypic and genotypic diversity, antibiotic resistance, frequency of virulence genes and genome sequencing in *Pseudomonas aeruginosa* strains isolated from lobster. Int. J. Food Microbiol..

[32] Matloko K., Fri J., Ateba T.P., Molale-Tom L.G., Ateba C.N. (2021). Evidence of potentially unrelated AmpC beta-lactamase producing Enterobacteriaceae from cattle, cattle products and hospital environments commonly harboring the *bla*_ACC_ resistance determinant. PLoS ONE.

[33] Yaici L., Haenni M., Métayer V., Saras E., Mesbah Zekar F., Ayad M., Touati A., Madec J.-Y. (2017). Spread of ESBL/AmpC-producing *Escherichia coli* and *Klebsiella pneumoniae* in the community through ready-to-eat sandwiches in Algeria. Int. J. Food Microbiol..

[34] Vu T.T.T., Alter T., Roesler U., Roschanski N., Huehn S. (2018). Investigation of extended-spectrum and AmpC β-lactamase–producing Enterobacteriaceae from retail seafood in Berlin, Germany. J. Food Prot..

[35] 2013/652/EU: Commission Implementing Decision of 12 November 2013 on the Monitoring and Reporting of Antimicrobial Resistance in Zoonotic and Commensal Bacteria. http://data.europa.eu/eli/dec_impl/2013/652/o.

[36] 2020/1729/EU: Commission Implementing Decision (EU) 2020/1729 of 17 November 2020 on the Monitoring and Reporting of Antimicrobial Resistance in Zoonotic and Commensal Bacteria and Repealing Implementing Decision 2013/652/EU. http://data.europa.eu/eli/dec_impl/2020/1729/o.

[37] Roschanski N., Fischer J., Guerra B., Roesler U. (2014). Development of a multiplex Real-Time PCR for the rapid detection of the predominant beta-lactamase genes CTX-M, SHV, TEM and CIT-Type AmpCs in Enterobacteriaceae. PLoS ONE.

[38] Pérez-Pérez F.J., Hanson N.D. (2002). Detection of plasmid-mediated AmpC β-lactamase genes in clinical isolates by using multiplex PCR. J. Clin. Microbiol..

[39] Rodríguez I., Barownick W., Helmuth R., Mendoza M.C., Rodicio M.R., Schroeter A., Guerra B. (2009). Extended-spectrum ß-lactamases and AmpC ß-lactamases in ceftiofur-resistant *Salmonella enterica* isolates from food and livestock obtained in Germany during 2003–2007. J. Antimicrob. Chemother..

[40] Hammerl J.A., Klein I., Lanka E., Appel B., Hertwig S. (2008). Genetic and functional properties of the self-transmissible *Yersinia enterocolitica* plasmid pYE854, which mobilizes the virulence plasmid pYV. J. Bacteriol..

[41] Deneke C., Brendebach H., Uelze L., Borowiak M., Malorny B., Tausch S.H. (2021). Species-specific quality control, assembly and contamination detection in microbial isolate sequences with AQUAMIS. Genes.

[42] Hallgren M.B., Overballe-Petersen S., Lund O., Hasman H., Clausen P.T.L.C. (2021). MINTyper: An outbreak-detection method for accurate and rapid SNP typing of clonal clusters with noisy long reads. Biol. Methods Protoc..

[43] Zhou Z., Alikhan N.-F., Mohamed K., Fan Y., Achtman M. (2020). The EnteroBase user’s guide, with case studies on *Salmonella* transmissions, *Yersinia pestis* phylogeny, and *Escherichia* core genomic diversity. Genome Res..

[44] Clausen P.T.L.C., Aarestrup F.M., Lund O. (2018). Rapid and precise alignment of raw reads against redundant databases with KMA. BMC Bioinform..

[45] Deneke C., Uelze L., Brendebach H., Tausch S.H., Malorny B. (2021). Decentralized investigation of bacterial outbreaks based on hashed cgMLST. Front. Microbiol..

[46] European Food Safety Authority, European Centre for Disease Prevention and Control (2023). The European Union Summary Report on Antimicrobial Resistance in zoonotic and indicator bacteria from humans, animals and food in 2020/2021. EFSA J..

[47] Mattioni Marchetti V., Bitar I., Mercato A., Nucleo E., Marchesini F., Mancinelli M., Prati P., Scarsi G.S., Hrabak J., Pagani L. (2020). Deadly puppy infection caused by an MDR *Escherichia coli* O39 *bla*_CTX–M–15_, *bla*_CMY–2_, *bla*_DHA–1_, and *aac(6)-Ib-cr* positive in a breeding kennel in Central Italy. Front. Microbiol..

[48] Clément M., Keller P.M., Bernasconi O.J., Stirnimann G., Frey P.M., Bloemberg G.V., Sendi P., Endimiani A. (2019). First clinical case of in vivo acquisition of DHA-1 plasmid-mediated AmpC in a *Salmonella enterica* subsp. *enterica* isolate. Antimicrob. Agents Chemother..

[49] Juraschek K., Borowiak M., Tausch S.H., Malorny B., Käsbohrer A., Otani S., Schwarz S., Meemken D., Deneke C., Hammerl J.A. (2021). Outcome of different sequencing and assembly approaches on the detection of plasmids and localization of antimicrobial resistance genes in commensal *Escherichia coli*. Microorganisms.

[50] Hennequin C., Ravet V., Robin F. (2018). Plasmids carrying DHA-1 β-lactamases. Eur. J. Clin. Microbiol. Infect. Dis..

[51] Mahrouki S., Chihi H., Bourouis A., Ayari K., Ferjani M., Moussa M.B., Belhadj O. (2015). Nosocomial dissemination of plasmids carrying *bla*_TEM-24_, *bla*_DHA-1_, *aac(6’)-Ib-cr*, and *qnrA6* in *Providencia* spp. strains isolated from a Tunisian hospital. Diagn. Microbiol. Infect. Dis..

[52] Harmer C.J., Hall R.M. (2016). IS 26 -mediated formation of transposons carrying antibiotic resistance genes. mSphere.

[53] Harmer C.J., Moran R.A., Hall R.M. (2014). Movement of IS 26-associated antibiotic resistance genes occurs via a translocatable unit that includes a single IS26 and preferentially inserts adjacent to another IS26. mBio.

[54] Harmer C.J., Hall R.M. (2015). IS26-mediated precise excision of the IS26-aphA1a translocatable unit. mBio.

[55] Cain A.K., Liu X., Djordjevic S.P., Hall R.M. (2010). Transposons related to Tn 1696 in IncHI2 plasmids in multiply antibiotic resistant *Salmonella enterica* serovar Typhimurium from Australian animals. Microb. Drug Resist..

