# Supplementary material for: Rare Plasmid-Mediated AmpC Beta-Lactamase DHA-1 Located on Easy Mobilized IS26-Related Genetic Element Detected in Escherichia coli from Livestock and Food in Germany
Source: Microorganisms. 2024 Mar 21;12(3):632. doi: 10.3390/microorganisms12030632 (PMC10975689; doi:10.3390/microorganisms12030632)
Supplement: Supplementary file 1 [file microorganisms-12-00632-s001.zip › microorganisms-2874017-supplementary.pdf]

Table S1. SNPs analysis of DHA-1 producing *E. coli* from the food chain in Germany using MINTyper (Malte B Hallgren et al., 2021) with default settings. Unicycler hybrid-assembly of 17-AB00899 was used as reference genome.

ID SAMPLES	17-AB00899	19-AB00708	19-AB01177	19-AB01178	19-AB01179	19-AB01500	19-AB02469	21-AB01800
**17-AB00899**	0							
**19-AB00708**	11036	0						
**19-AB01177**	11281	1076	0					
**19-AB01178**	21178	22975	23144	0				
**19-AB01179**	11567	4100	4355	23007	0			
**19-AB01500**	29130	31330	31398	32833	31356	0		
**19-AB02469**	34200	36512	36413	37838	36573	37702	0	
**21-AB01800**	9294	12813	12753	22936	13305	31178	35994	0

References

1. Hallgren, M.B.; Overballe-Petersen, S.; Lund, O.; Hasman, H.; Clausen, P. T. L. C.; MINTyper: an outbreak-detection method for accurate and rapid SNP typing of clonal clusters with noisy long reads, *Biol Methods Prot* Volume 6, Issue 1, **2021,** bpab008, https://doi.org/10.1093/biomethods/bpab008
